# The plastidial metabolite 2‐*C*‐methyl‐*D*‐erythritol‐2,4‐cyclodiphosphate modulates defence responses against aphids

**DOI:** 10.1111/pce.13538

**Published:** 2019-03-08

**Authors:** Nawaporn Onkokesung, Michael Reichelt, Louwrance P. Wright, Michael A. Phillips, Jonathan Gershenzon, Marcel Dicke

**Affiliations:** ^1^ Laboratory of Entomology Wageningen University Wageningen The Netherlands; ^2^ Department of Biochemistry Max Planck Institute for Chemical Ecology Jena Germany; ^3^ Department of Biology and Graduate Program in Cellular and Systems Biology University of Toronto‐Mississauga Mississauga Ontario Canada

**Keywords:** aphid resistance, Arabidopsis, indole glucosinolates, phloem‐sucking herbivores, phytohormone signalling, retrograde signalling, secondary metabolites

## Abstract

Feeding by insect herbivores such as caterpillars and aphids induces plant resistance mechanisms that are mediated by the phytohormones jasmonic acid (JA) and salicylic acid (SA). These phytohormonal pathways often crosstalk. Besides phytohormones, methyl‐*D*‐erythriol‐2,4‐cyclodiphosphate (MEcPP), the penultimate metabolite in the methyl‐*D*‐erythritol‐4‐phosphate pathway, has been speculated to regulate transcription of nuclear genes in response to biotic stressors such as aphids. Here, we show that MEcPP uniquely enhances the SA pathway without attenuating the JA pathway. Arabidopsis mutant plants that accumulate high levels of MEcPP (*hds3*) are highly resistant to the cabbage aphid (*Brevicoryne brassicae*), whereas resistance to the large cabbage white caterpillar (*Pieris brassicae*) remains unaltered. Thus, MEcPP is a distinct signalling molecule that acts beyond phytohormonal crosstalk to induce resistance against the cabbage aphid in Arabidopsis. We dissect the molecular mechanisms of MEcPP mediating plant resistance against the aphid *B*. *brassicae*. This shows that MEcPP induces the expression of genes encoding enzymes involved in the biosynthesis of several primary and secondary metabolic pathways contributing to enhanced resistance against this aphid species. A unique ability to regulate multifaceted molecular mechanisms makes MEcPP an attractive target for metabolic engineering in *Brassica* crop plants to increase resistance to cabbage aphids.

## Abstract 2

The plastidial metabolite methyl‐*D*‐erythriol‐2,4‐cyclodiphosphate is a positive regulator of genes in SA signalling and biosynthetic genes in various metabolic pathways that confer resistance to aphids without compromising resistance to caterpillars.

## 1 INTRODUCTION

Approximately half a million insect herbivore species are known, and phloem‐sucking species including aphids are among the major causes of yield losses in agriculture around the world (Oerke, 2006). As a feeding strategy, aphids utilize delicate stylets for manoeuvering around mesophyll cells and then penetrate phloem cells to feed on the phloem sap (Tjallingii & Hogen Esch, 1993; Walling, 2000). Although aphid infestation does not inflict major physical damage to host plants, it reduces host‐plant productivity due to a significant loss of nutrients (Girousse, Moulia, Silk, & Bonnemain, 2005; Lemoine et al., 2013). Furthermore, many aphid species are known to transmit viral diseases to host plants, which will further hamper plant productivity (Ng & Perry, 2004). Chemical insecticides have been widely used to control aphids on *Brassica* crop plants such as oilseed rape. The use of insecticides has led to the development of insecticide resistance in aphid populations. For instance, there has been a significant increase in insecticide‐resistant peach‐potato aphids in the United Kingdom (Bass et al., 2014; Foster, Denholm, & Devonshire, 2000). Furthermore, new European Union legislation has recently banned several insecticides, which consequently creates significant constraints on pest management in agricultural systems. Therefore, new strategies to improve crop protection against insect herbivores, especially aphids, are urgently required to prevent the potentially devastating impacts of aphid infestation on crop production. To achieve this goal, the understanding of molecular mechanisms of host‐plant resistance against aphids will provide fundamental knowledge needed for developing new classes of chemicals or breeding plants for enhanced resistance against aphids.

To date, the understanding of plant resistance to aphids mostly derives from studies on *Arabidopsis thaliana*. Plant hormones including salicylic acid (SA), jasmonates (JAs), and their interactions are well known to facilitate and tailor plant responses against various aphid species (de Vos, Kim, & Jander, 2007; Erb, Meldau, & Howe, 2012; Kloth et al., 2016; Mewis, Appel, Hom, Raina, & Schultz, 2005). For instance, Arabidopsis mutant plants containing a nonfunctional JA receptor protein, CORONATINE INSENSITIVE 1 (COI1), are highly susceptible to the cabbage aphid *Brevicoryne brassicae* and the green peach aphid *Myzus persicae* (Mewis et al., 2005). Furthermore, a mutation in *NONEXPRESSOR OF PR gene 1* (*NPR1*), a gene coding for an important SA receptor protein in Arabidopsis, rendered mutant plants more susceptible to *B. brassicae* aphids (Mewis et al., 2005). Transcript and metabolite studies in these Arabidopsis mutants showed that JA, SA, and their crosstalk are involved in the regulatory network underlying the biosynthesis of plant defence metabolites against aphids including glucosinolates and camalexin (Kim & Jander, 2007; Kuśnierczyk et al., 2008; Mewis et al., 2005).

**Figure 1:**
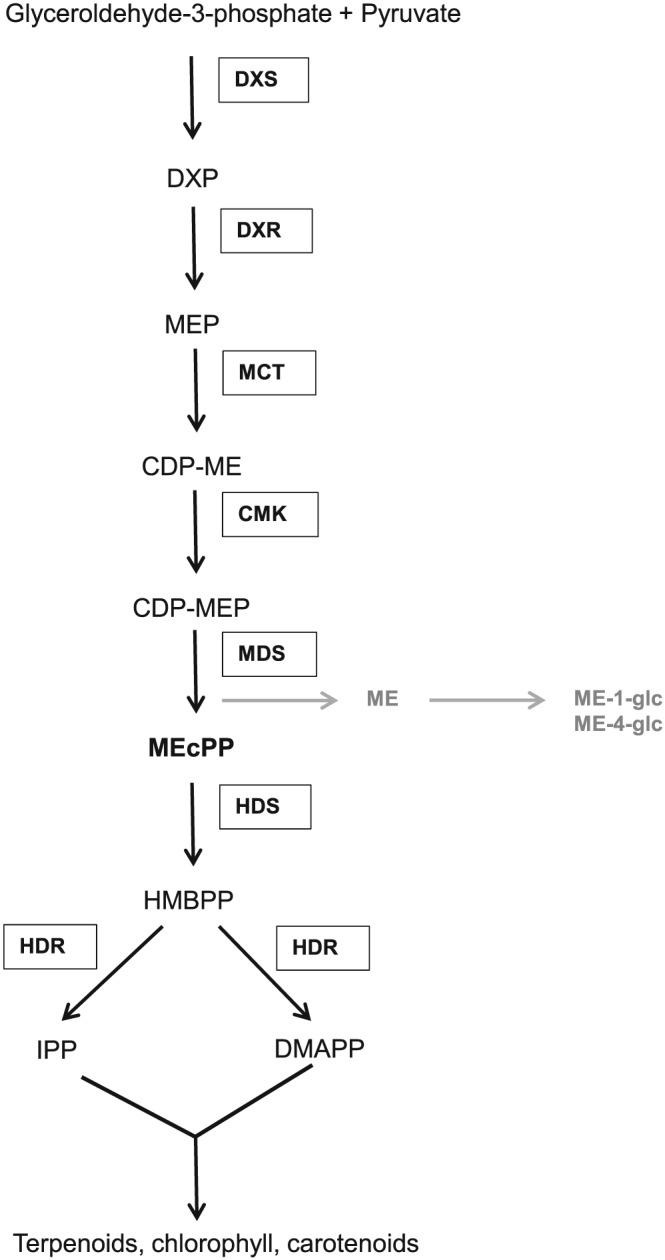
Scheme of methyl‐*D*‐erythritol‐4‐phosphate (MEP) pathway in *Arabidopsis thaliana*. The main MEP pathway is indicated in black. The MEP‐related pathway is indicated in grey. Abbreviation: DXS, 1‐deoxy‐*D*‐xylulose‐5‐phosphate synthase; DXP, 1‐deoxy‐*D*‐xylulose 5‐phosphate; DXR, 1‐deoxy‐*D*‐xylulose 5‐phosphate reductoisomerase; MEP, methyl‐*D*‐erythritol‐4‐phosphate; MCT, 2‐*C*‐ methyl‐*D*‐erythritol‐4‐phosphate cytidylytransferase; CDP‐ME, 4‐(cytidine 5′‐diphospho)‐2‐*C*‐methyl‐*D*‐erythritol; CMK, 4‐(cytidine 5′‐diphospho)‐2‐*C*‐methyl‐*D*‐erythritol kinase; CDP‐MEP, 4‐(cytidine 5′‐diphospho)‐2‐*C*‐methyl‐*D*‐erythritol‐2,4‐cyclodiphosphate; MDS, 2‐*C*‐methyl‐*D*‐erythritol‐2,4‐cyclodiphosphate synthase; MEcPP, 2‐*C*‐methyl‐*D*‐erythritol‐2,4‐cyclodiphosphate; HDS, 2‐(*E*)‐4‐hydroxy‐3‐methylbut‐2‐enyl diphosphate synthase; HMBPP, 2‐(*E*)‐4‐hydroxy‐3‐methylbut‐2‐enyl diphosphate; HDR, (*E*)‐4‐hydroxy‐3‐methylbut‐2‐enyl diphosphate reductase; IPP, isopentenyl diphosphate; DMAPP, dimethylallyl diphosphate; ME, methyl‐*D*‐erythritol, ME‐1‐glc, 2‐*C*‐methyl‐*d*‐erythritol‐*O*‐1‐β‐*d*‐glucopyranoside, ME‐4‐glc, 2‐*C*‐methyl‐*d*‐erythritol‐*O*‐4‐β‐*d*‐glucopyranoside

Apart from plant hormones, low‐molecular weight metabolites such as trehalose‐6‐phosphate (T6P) and 2‐*C*‐methyl‐*D*‐erythriol‐2,4‐cyclodiphosphate (MEcPP) have recently been proposed to function as signalling molecules in plant responses to environmental stresses (González‐Cabanelas et al., 2015; Griffiths et al., 2016; Singh, Louis, Ayre, Reese, & Shah, 2011). T6P and trehalose play a role in sugar signalling that mediates responses to (a)biotic stresses as well as growth and development in Arabidopsis and spring wheat (Griffiths et al., 2016; Schluepmann, Pellny, van Dijken, Smeekens, & Paul, 2003; Singh et al., 2011). However, the role of MEcPP as a plastid‐to‐nucleus retrograde signalling molecule that triggers transcriptional reprogramming of genes involved in plant stress responses has remained inconclusive to date. As the penultimate metabolite in the methyl‐*D*‐erythritol‐4‐phosphate (MEP) pathway, MEcPP is converted by 2‐(*E*)‐4‐hydroxy‐3‐methylbut‐2‐enyl diphosphate synthase (HDS) to (2*E*)‐4‐hydroxy‐3‐methylbut‐2‐enyl diphosphate (HMBPP). Then (*E*)‐4‐hydroxy‐3‐methylbut‐2‐enyl diphosphate reductase (HDR) converts HMBPP into isopentenyl diphosphate (IPP) and dimethylallyl diphosphate (DMAPP), the universal precursors of plant isoprenoids, including carotenoids and chlorophylls (Figure 1; Kirby & Keasling, 2009; Phillips, Leon, Boronat, & Rodriguez‐Concepcion, 2008). A point mutation leading to changes in the HDS protein at leucine^703^ or glycine^696^ renders two independent Arabidopsis mutant lines, namely, *ceh1* and *hds3*, respectively that accumulate constitutively high levels of MEcPP (Gil, Coego, Mauch‐Mani, Jorda, & &Vera P., 2005; Xiao et al., 2012). It is interesting that these two mutant lines are highly resistant to biotrophic pathogens and cabbage aphids (Gil et al., 2005; González‐Cabanelas et al., 2015; Xiao et al., 2012). Furthermore, an increased transcript expression of genes in the SA biosynthetic and responsive pathways in *ceh1* and *hds3* mutant plants suggests a role of MEcPP as a positive regulator of the SA‐signalling pathway, mediating plant resistance to biotrophic pathogens and aphids (Gil et al., 2005; González‐Cabanelas et al., 2015; Xiao et al., 2012). In recent years, evidence supporting a function of MEcPP as a signalling molecule mediating plant responses to (a)biotic stresses has been presented (Gil et al., 2005; González‐Cabanelas et al., 2015; Lemos et al., 2016; Walley et al., 2015; Xiao et al., 2012). However, the mechanisms underlying MEcPP functions in mediating transcriptional reprogramming of genes involved in plant stress responses has remained inconclusive to date.

Plant defence responses to caterpillar feeding are mediated by the JA‐signalling pathway (Reymond et al., 2004), and the antagonistic crosstalk of the SA and JA‐signalling pathways is known to influence plant responses to caterpillar feeding (Mewis et al., 2005; Kroes, van Loon, & Dicke, 2015; Walling, 2008). For instance, the Arabidopsis T‐DNA‐insertion mutant plants that are silenced in the expression of the gene encoding the WRKY70 transcription factor (*wrky70*), an important transcription regulator involved in antagonistic effects of SA on the JA‐signalling pathway, are resistant to *P. brassicae* caterpillars (Onkokesung, Reichelt, van Doorn, Schuurink, & Dicke, 2016). Furthermore, high relative transcript expression of JA‐responsive genes corresponds with increased resistance to *P. brassicae* caterpillar in *wrky70* mutants (Onkokesung et al., 2016). Because transcription of genes in the SA‐signalling pathway is constitutively high in *hds3* plants, the question arises whether MEcPP and the robust SA‐signalling pathway in *hds3* plants could attenuate the JA‐signalling pathway and JA‐induced resistance to *P. brassicae* caterpillars.

The present study aims to gain detailed insight into the molecular mechanisms from signalling to biosynthesis of defence metabolites underlying the effects of MEcPP on resistance to the cabbage aphid (*B. brassicae*). Furthermore, the impacts of MEcPP on antagonistic interactions of the SA‐ and JA‐signal transduction pathways that could affect JA‐induced resistance to the cabbage white caterpillar *P. brassicae* have also been investigated. Bioassays assessing caterpillar or aphid performance in combination with transcript and metabolite analyses to assess the effects of MEcPP on molecular mechanisms of plant responses to aphid or caterpillar feeding have been conducted in MEcPP‐accumulation mutant (*hds3*) plants. These analyses have been complemented with investigations of a mutant overexpressing *HDS* in the *hds3* background: 35S:HDS (*hds3*).

## 2 MATERIALS AND METHODS

### 2.1 Plant material and growth conditions

*A. thaliana* ecotype Columbia‐0 (Col‐0) was used as wild type (WT) reference. The *hds3* (formerly *csb3*) Arabidopsis ethyl methanesulfonate mutation line and the HDS complemented mutant (35S:HDS, *hds3*) have been previously described (Gil et al., 2005). Seeds were surface sterilized and washed three times in sterile deionized water before germination on half‐strength Murashige and Skoog (MS) medium containing 3% sucrose. Plates were stratified for 2 days before transferring to a growth chamber at 21 ± 1°C, 60 ± 5% relative humidity (RH), 120 μmol m^−2^ s^−1^ light intensity, and 8:16 (light:dark) photoperiod. Fourteen‐day‐old seedlings were subsequently transplanted into round plastic pots (4.5‐cm diameter) containing sterilized substrate mix (Horticoop, the Netherlands) and kept under environmental conditions as described above. Four‐ to 5‐week‐old plants were used in all experiments.

### 2.2 Insects

Caterpillars (*P. brassicae*) and aphids (*B. brassicae*) from their respective stock colonies (Laboratory of Entomology, Wageningen University, the Netherlands) were reared on Brussels sprouts (*Brassica oleracea var. gemmifera* cv Cyrus) at 22 ± 1°C, 60 ± 5% RH, and 16:8 (light: dark) photoperiod.

### 2.3 Herbivore performance

Four‐ to 5‐week‐old Arabidopsis plants were transferred from short (8:16 light: dark) to long day (16:8 light: dark) photoperiod conditions, at 21 ± 1°C, 60 ± 5% RH, and plants were kept at long day conditions for 48 hr before herbivore treatments.

For *B. brassicae* aphids, two first‐instar aphid nymphs were placed on an individual WT, *hds3*, or 35S:HDS (*hds3*) plant. Twenty plants from each plant type were used. Plants were kept individually in cylindrical plastic containers (8‐cm diameter × 14‐cm height) covered with fine mesh gauze under long‐day photoperiod conditions. The aphid nymphs fed freely on the plants. After 7 days of feeding (time to reach the adult stage), each plant was examined, and one adult aphid was removed if both aphids had survived. The remaining single adult aphid was kept under the same conditions for another 7 days before the total number of aphids on each individual plant was counted. The number of aphids per plant (*n* = 20) was compared among plant types.

For *P. brassicae* caterpillars, a freshly hatched neonate caterpillar was placed on a WT, *hds3*, or 35S:HDS (*hds3*) plant (one caterpillar per plant). Twenty plants from each plant type were used. The caterpillars fed freely on the plants. Fresh body mass of individual caterpillars was measured at 4, 7, and 9 days of feeding on WT, *hds3*, or 35S:HDS (*hds3*) plants. Caterpillar body mass (*n* = 20) was compared among plant types at each time point.

### 2.4 Aphid treatment

For primary and secondary plant metabolite analysis, *B. brassicae* nymphs were placed on individual WT, *hds3*, or 35S:HDS (*hds3*) plants: one nymph per plant. Ten plants from each plant type were used. Control (undamaged) plants received no aphids. Plants were kept in cylindrical plastic containers covered with mesh gauze at 16 L:8D photoperiod, 21 ± 1°C, and 60 ± 5% RH conditions. Aphids fed freely on the Arabidopsis plants. After 7 days of feeding, aphids were removed, and aerial plant tissues were harvested and pooled from two individual plants to obtain one biological replicate. Tissue samples were flash‐frozen in liquid nitrogen and stored at −80°C until analysis.

For transcript analysis of plants, 20 first‐instar *B. brassicae* nymphs were placed on each individual WT, *hds3*, or 35S:HDS (*hds3*) plant. Plants were kept in closed containers as described above. Aerial plant tissues from two individual plants were harvested after 6, 24, 48, and 72 hr of aphid feeding and pooled to obtain one biological replicate. Five biological replicates were used for each time point. Tissue samples were immediately frozen in liquid nitrogen and stored at −80°C until analysis.

### 2.5 Analysis of MEP pathway metabolites

Isoprenoid metabolites including 1‐deoxy‐*D*‐xylulose 5‐phosphate (DXP), MEcPP, IPP, and DMAPP were extracted as described by González‐Cabanelas, Hammerbacher, Raguschke, Gershenzon, and Wright (2016). Briefly, approximately 5 mg of lyophilized plant material was extracted twice with 250 μl 50% acetonitrile containing 10 mM of ammonium acetate (pH 9.0). Plant extracts were centrifuged, and the supernatants were air dried under nitrogen gas at 40°C. Samples were dissolved in 100 μl of 10 mM of ammonium acetate (pH 9.0), and 100 μl of chloroform was added to remove lipids. The upper aqueous phase was transferred to a 1.5 ml of Eppendorf tube and diluted 1:1 (*v*/*v*) with acetonitrile. For ME‐glucose extraction, approximately 5 mg of lyophilized tissue was extracted in 500 μl of 60% methanol. The plant extracts were centrifuged at 16,000 *g* for 5 min. The supernatant was used for analysis.

Isoprenoid metabolites were analysed on an Agilent 1260 Infinity HPLC system (Agilent Technologies, Germany) connected to an API 5000 triple quadrupole mass spectrometer (Applied Biosystems, Germany). DXP, MEcPP, and IPP/DMAPP (eluting as a single peak) were separated on an XBridge Amide column (3.5 μm, 150 × 2.1 mm, Waters, Germany) with an HILIC guard column containing the same sorbent (3.5 μm, 10 × 2.1 mm) and an SSI™ high pressure precolumn filter (Sigma) as described in González‐Cabanelas et al. (2016). The isoprenoid metabolites were quantified using external standard curves containing added internal standards (ISTD) consisting of [^13^C]DXP, MEcPP, IPP, and DMAPP as described in González‐Cabanelas et al. (2016).

### 2.6 Glucosinolate analysis

Approximately 20 mg of lyophilized tissue was used for glucosinolate extraction and analyzed by high‐performance liquid chromatography and UV detection; glucosinolates from the 80% methanol (*v*:*v*) extracts were bound to DEAE‐Sephadex and converted to desulfoglucosinolates by the use of *Helix pomatia* sulfatase (Burow, Muller, Gershenzon, & Wittstock, 2006). An HPLC instrument (Agilent 1100 Series), equipped with a C‐18 reverse phase column (Nucleodur Sphinx RP, 250 × 4.6 mm, 5‐μm particle size, Macherey‐Nagel, Germany), was used as described by Burow et al. (2006). Desulfoglucosinolates were identified based on comparison of retention times and UV absorption spectra with those of known standards. Glucosinolate levels (μmol g^−1^ dry weight) were calculated from the peak areas at 229 nm relative to the peak area of the internal standard *para*‐hydroxybenzyl glucosinolate using the relative response factor 2.0 for aliphatic and 0.5 for indolic glucosinolates (Burow et al., 2006; Onkokesung et al., 2014).

### 2.7 Biological activity of MEcPP‐ and MEcPP‐related metabolites

Two leaves of 4‐week‐old WT, *hds3*, or 35S:HDS (*hds3*) plants were wounded by ultrafine tweezers. A solution of MEcPP or a mixture of MEcPP and ME (MEcPP + ME) at 100 μg ml^−1^ in 0.02% Silwet‐77 was exogenously applied on both sides of the wounded leaves with a fine brush. Plants were kept in the growth chamber for 2 hr before harvesting. Two wounded leaves were pooled to obtain one biological replicate. Three biological replicates were harvested from control, wounded, or exogenous application treatment plants. Leaf tissues were flash‐frozen in liquid nitrogen and kept at −80°C until used for RNA isolation followed by cDNA synthesis.

### 2.8 Quantitative real‐time PCR

Approximately 100 mg of finely ground frozen leaf tissue was used for total RNA isolation using NucleoSpin RNA plant kit (Macherey‐Nagel, Germany). DNA residues were removed from total RNA samples via RQ1 DNase treatment (Promega, the Netherlands), followed by ethanol precipitation. One micorgram of RNA was used for cDNA synthesis using an iScript cDNA synthesis kit for RT‐qPCR (Bio‐Rad, the Netherlands) in a 20‐μl reaction volume. Quantitative real‐time PCR was performed in a CFX96 Touch™ Real Time PCR Detection System (Bio‐Rad, the Netherlands) in a total volume of 20 μl containing 1.5 μl of cDNA from 1 μg of RNA, 10 μl of iQ SYBR green supermix (Bio‐Rad, the Netherlands), and 1.2 μl of 5 μM forward and reverse gene‐specific primers. The primer sequences used in this study are listed in Table S1. The reactions were run in a three‐step programme including melting curve analysis, initial incubation at 95°C for 10 min, followed by amplification for 40 cycles (95°C for 15 s, 59°C for 30 s, and 72°C for 45 s), and melting curve analysis from 72°C to 95°C. The specific primers of elongation factor‐1α from Arabidopsis (AtEF‐1α; accession NM_001125992) were used for normalization. Relative transcript expression (fold‐change) was calculated based on an efficiency corrected model (Pfaffl, 2001). Mean relative transcript expression from five biological replicates was used for statistical analysis.

### 2.9 Data analysis

All data were analysed by SPSS version 24 software (IBM, Chicago, IL, USA). Data on herbivore performance, transcript expression, and metabolite analysis were analysed by Student's *t* test or one‐way analysis of variance (ANOVA) followed by posthoc test. The effect of plant types and treatment conditions on specific aliphatic or indolic GLS in Figure 3 were analysed by two‐way ANOVA. The assumption of homogeneity of variance for one‐way and two‐way ANOVA was tested by Levene's test.

## 3 RESULTS

### 3.1 Differential effects of MEcPP accumulation on plant resistance against aphids *(* *B. brassicae*) or caterpillars (*P. brassicae*)

**Figure 2:**
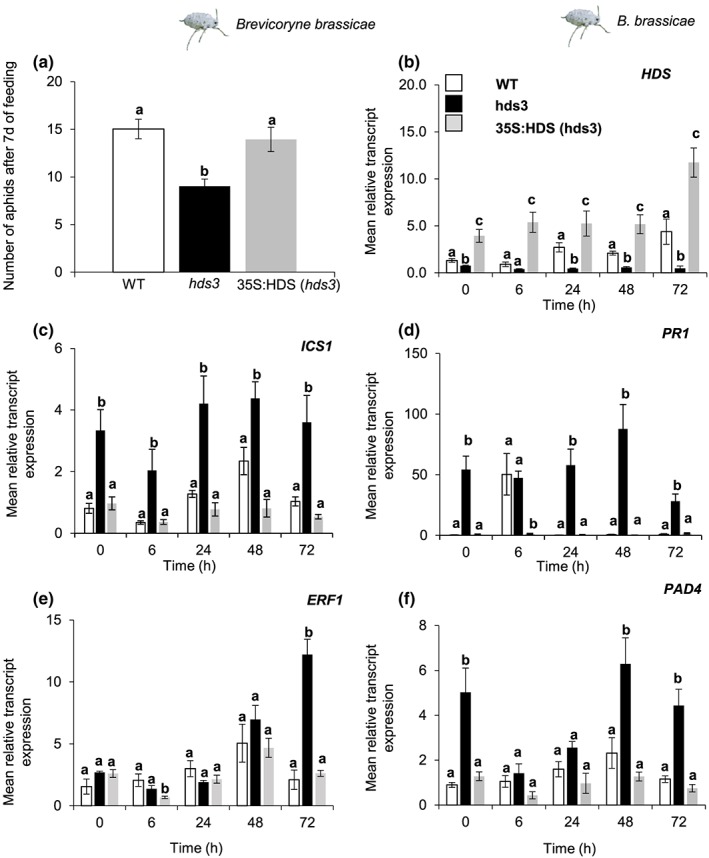
Effects of MEcPP on plant resistance to the cabbage aphid *Brevicoryne brassicae* in Arabidopsis. Impact of MEcPP on plant resistance to the cabbage aphid was determined by bioassay and the relative transcript expression of selected genes. (a) Average of aphid progeny (mean ± *SE*; *n* = 20) after 9 days of feeding on WT, *hds3*, or 35S:HDS (*hds3)* plants. The kinetics of relative transcript expression of (b) *HDS*, (c) the SA biosynthetic marker gene *ICS1*, (d) the SA‐responsive marker gene *PR1*, and (e–f) genes induced by *B. brassicae* aphid infestation (*ERF* and *PAD4*) from leaf tissue of each plant type. Values represent average expression ± *SE* (*n* = 5) of each gene relative to the relative transcript expression of the *ELONGATION FACTOR‐1a* (*EF1a*) gene. Aphid progeny and relative transcript expression were compared among WT, *hds3*, and 35S:HDS (*hds3*) plants by one‐way ANOVA followed by Tukey's honestly significant difference (HSD) posthoc test. Different letters indicate significant differences among plant genotypes for each time point separately (*P* ≤ 0.05) [Colour figure can be viewed at http://wileyonlinelibrary.com]

Using Arabidopsis mutant plants accumulating high levels of MEcPP (*hds3*), we recorded positive impacts of MEcPP on expression levels of genes in SA biosynthetic (*ISOCHORISMATE SYNTHASE1*, *ICS1*), SA responsive (*PATHOGENESIS‐RELATED 1, PR1*), and SA‐inducible (*PHYTOALEXIN DEFICIENT 4*, *PAD4*) pathways (Figure 2). Furthermore, an elevated expression of these genes coincided with enhanced resistance to the *B. brassicae* aphids in *hds3* mutant plants compared with WT plants (Figure 2a; González‐Cabanelas et al., 2015). Because the SA‐signalling pathway is known to suppress the JA‐signalling pathway (Kroes et al., 2015; Mewis et al., 2005; Walling, 2008), we probed the impacts of a robust SA‐signalling pathway in *hds3* mutant plants on JA‐signalling pathway by assessing JA‐induced plant responses to cabbage white caterpillars (*P. brassicae*). We quantified caterpillar performances and transcript expression of selected genes in the SA‐ and JA‐signalling pathways in three plant genotypes, namely, Arabidopsis ecotype Columbia‐0 (WT), the *hds3* mutant, and the complemented *hds3* mutant overexpressing *HDS* under the control of the CaMV 35S promoter in plants with an *hds3* mutant background (35S:HDS, *hds3*). In addition, we also assessed the transcript expression of *HDS* to examine whether caterpillar feeding affected *HDS* expression.

**Figure 3:**
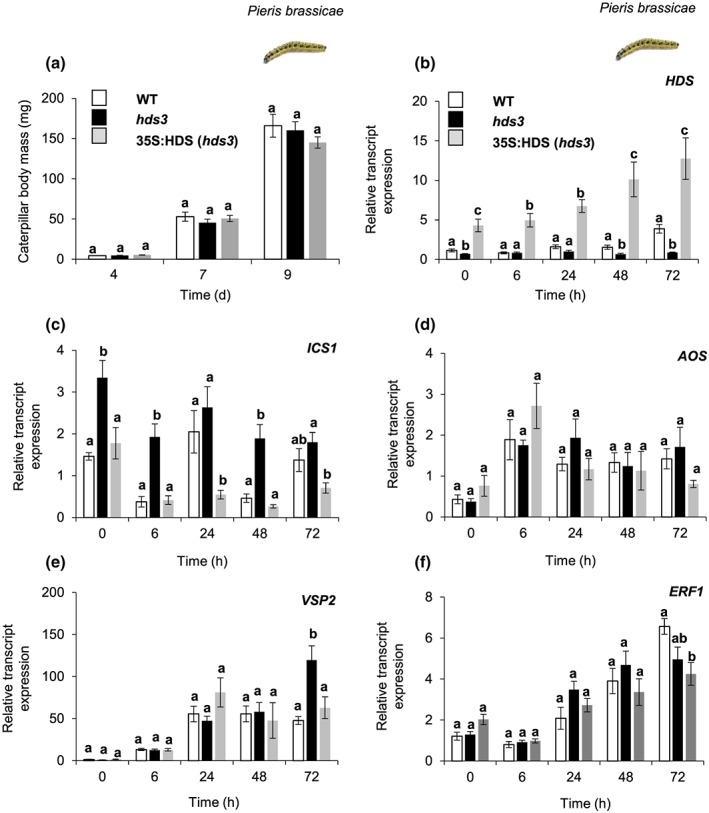
Plant resistance against the specialist caterpillar *Pieris brassicae*, and relative transcript expression of selected genes in phytohormonal signalling pathways. (a) Fresh body mass (mean ± *SE*; *n* = 20) of *P. brassicae* caterpillars after 4, 7, and 9 days of feeding on WT, *hds3*, or 35S:HDS (*hds3)* plants. Time course of expression of (b) *HDS*, (c) a salicylic acid (SA) biosynthetic gene (*ICS1*), (d) jasmonic acid (JA) biosynthetic gene (*AOS*), (e) a JA‐responsive marker gene (*VSP2*), and (f) a JA‐inducible transcription factor gene (*ERF1*). The value at each time point represents mean ± *SE* (*n* = 5) of *HDS*, *ICS1*, *AOS*, *VSP2*, and *ERF1* transcript levels relative to those of the *ELONGATION FACTOR‐1a* (*EF1a)* gene. Body mass and transcript expression were compared among WT, *hds3*, and 35S:HDS (*hds3*) plants by one‐way ANOVA followed by Tukey's honestly significant difference (HSD) posthoc test. Different letters indicate significant differences among plant genotypes for each time point separately (*P* ≤ 0.05)

It was intriguing that caterpillar performance was not affected by an increase in the SA‐signalling pathway in *hds3* plants. In fact, the caterpillars reached similar body mass after feeding on WT, *hds3*, and the complemented *hds3* mutant (35S:HDS, *hds3*) plants at all three time points observed in this study (Figure 3a; one‐way ANOVA; *P*_(4d)_ = 0.37, *P*_(7d)_ = 0.37, *P*_(9d)_ = 0.37).

The relative transcript level of *HDS* was much higher in 35S:HDS (*hds3*) plants than in WT or *hds3* plants (Figure 3b). In WT and in 35S:HDS (*hds3*) plants, the relative transcript level of the *HDS* gene remained unchanged at early time points (0–48 hr) and significantly increased after 72 hr of *P. brassicae* caterpillar feeding (Figures 3b and S1; one‐way ANOVA; *P*_(72h‐WT plant)_ = 0.001, *P*_(72h‐35S:HDS [*hds3*] plant)_ = 0.01)_._ These results suggest a delayed effect of *P. brassicae* caterpillar feeding on *HDS* gene expression.

The relative transcript level of an SA‐biosynthetic gene, *ICS1*, was high in *hds3* plants compared with the expression in WT or 35S:HDS (*hds3*) plants after caterpillar feeding (Figure 3c). The relative transcript levels of a JA‐biosynthetic gene (*ALLENE OXIDE SYNTHASE; AOS*), a JA‐responsive marker gene (*VEGETATIVE STORAGE PROTEIN 2*; *VSP2*), and a JA‐regulated transcription factor (*ETHYLENE‐REPONSIVE ELEMENT‐BINDING PROTEIN 1; ERF1*; Lorenzo, Piqueras, Sánchez‐Serrano, & Solano, 2003) in *hds3* plants increased in a similar manner over time as in WT plants upon *P. brassicae* caterpillar feeding (Figure 3d–f). It is noteworthy that we observed a reduction in *ICS1* transcript expression at 6 hr of caterpillar feeding in *hds3* plants, while the expression of *AOS* was increased at the same time point (Figure 3c,d). These results suggest an antagonistic interaction of JA on SA biosynthetic pathways mediated by MEcPP. Together, these results indicate that a significant increase in relative expression of genes in the SA‐signalling pathway in *hds3* plants does not interfere with *P. brassicae* caterpillar‐induced expression of genes in the JA biosynthetic and responsive pathways. Overall, our results indicate the unique function of MEcPP as an enhancer of the SA‐signalling pathway and plant resistance against the *B. brassicae* aphid that does neither attenuate the JA‐signalling pathway nor plant resistance to *P. brassicae* caterpillars.

### 3.2 Feeding by *B. brassicae* aphids influences levels of MEcPP‐ and MEcPP‐related metabolites

**Figure 4:**
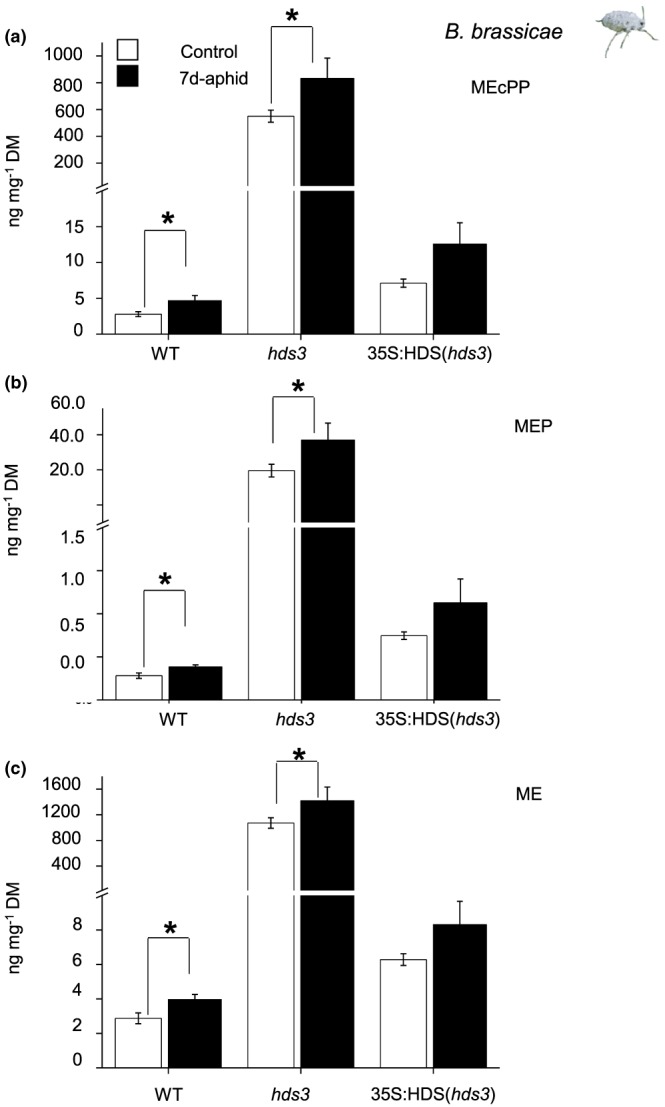
Feeding by *Brevicoryne brassicae* aphids induces MEcPP‐ and MEcPP‐related metabolite levels in Arabidopsis. The levels of (a) MEcPP, (b) MEP, and (c) ME were quantified in undamaged (control) and aphid‐damaged WT, *hds3*, and 35S:HDS (h*ds3*) leaves by HPLC/MS. The levels (mean ± *SE*; *n* = 5) of MEcPP‐ and MEcPP‐related metabolites were compared within each plant type by Student's *t* test; asterisks represent significant difference (*P* ≤ 0.05). Abbreviations: DM, dry matter; MEcPP, 2‐*C*‐methyl‐*D*‐erythritol‐2,4‐cyclodiphosphate; MEP, methyl‐*D*‐erythritol‐4‐phosphate; ME, methyl‐*D*‐erythritol [Colour figure can be viewed at http://wileyonlinelibrary.com]

As we confirmed a positive impact of MEcPP on plant resistance against the aphid *B. brassicae* (Figure 2), we further examined MEcPP accumulation in response to *B. brassicae* infestation. It has been previously reported that the constitutive level of MEcPP was immensely elevated (~100 fold) in *hds3* mutants. Our results confirm elevated levels of MEcPP, MEP, and their free tetraol form, methyl‐*D*‐erythritol (ME) in *hds3* mutants compared with WT plants. The levels of MEcPP and its related metabolites can be enhanced through complementation with a functional *HDS* gene in *hds3* mutant plants (Figure 4). Interestingly, feeding by *B. brassicae* aphids for 7 days significantly induced MEcPP, MEP, and ME levels from the constitutive levels in *hds3* plants (Figure 4; Student's *t* test; *P*_(MEcPP)_ = 0.001, *P*_(MEP)_ = 0.02, *P*_(ME)_ = 0.006). Moreover, aphid feeding also significantly induced MEcPP, MEP, and ME levels in WT plants (Figure 4; Student's *t* test; *P*_(MEcPP)_ = 0.04, *P*_(MEP)_ = 0.04, *P*_(ME)_ = 0.03). Similar trends in the accumulation of MEcPP‐ and MEcPP‐related metabolites after *B. brassicae* aphid feeding were also observed in 35S:HDS (*hds3*) plants. However, the induced levels of these metabolites were not significantly different from the constitutive levels in the complemented mutant plants (Figure 4; Student's *t* test; *P*_(MEcPP)_ = 0.108, *P*_(MEP)_ = 0.21, *P*_(ME)_ = 0.18). Interestingly, aphid feeding did not induce higher levels of the first metabolite (DXP), and the final products (IPP and DMAPP) of the MEP pathway in WT, *hds3*, and 35S:HDS (*hds3*) plants (Figure S2; one‐way ANOVA; *P*_(DXP‐aphid)_ = 0.10, *P*_(IPP + DMAPP‐aphid)_ = 0.20). Together, our results show that *B. brassicae* infestation specifically induces MEcPP and its closely related metabolites without affecting the levels of upstream or downstream metabolites in the MEP pathway.

### 3.3 High MEcPP level affects glucosinolate accumulation

Plant hormonal signalling, including SA signalling, has previously been reported to underlie the regulatory network of glucosinolate (GLS) biosynthesis in Arabidopsis (Mewis et al., 2005). Because of enhanced expression of *ICS1* and *PR1* in *hds3* plants (Figure 2), we investigated whether this affected GLS accumulation and resistance to aphids in *hds3* plants. The GLS (aliphatic and indolic) levels as well as the relative transcript levels of selected genes in GLS metabolism were analysed for leaf tissues of WT, *hds3*, and 35S:HDS (*hds3*) plants before and after 7 days of *B. brassicae* aphid feeding.

**Figure 5:**
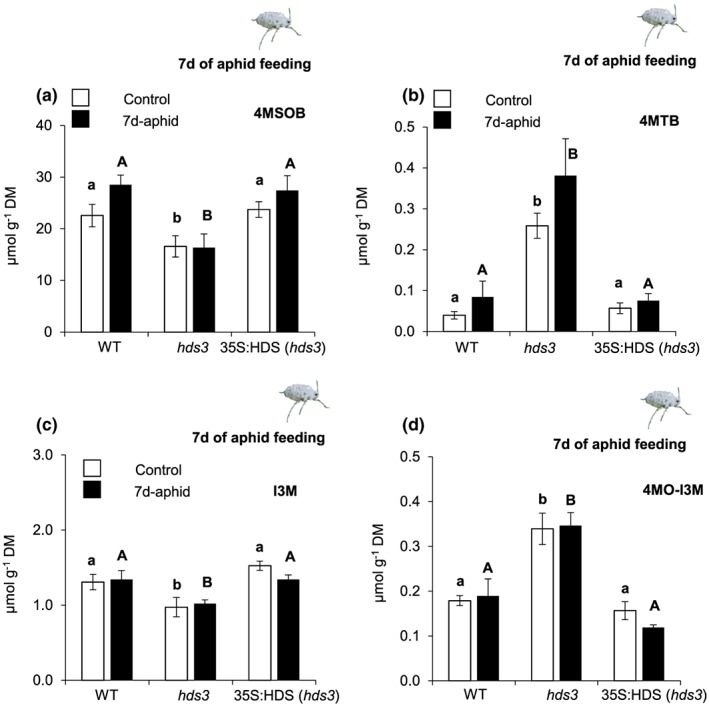
Levels of selected glucosinolates in WT, *hds3*, and 35S:HDS (*hds3*) leaves of uninfested control plants and plants infested with *Brevicoryne brassicae* aphids for 7 days. Aliphatic and indolic glucosinolates were quantified by HPLC/MS. Levels (means ± *SE*; *n* = 5) of (a) 4MSOB, (b) 4MTB, (c) I3M, and (d) 4MO‐I3M in undamaged leaves or leaves of plants after 7 days of aphid feeding. The levels of 4MSOB, 4MTB, I3M, and 4MO‐I3M were compared among WT, *hds3*, and 35S:HDS (*hds3*) plants by one‐way ANOVA followed by Tukey's honestly significant difference (HSD) posthoc test. Different letters indicate significant differences among plant genotypes, lower case letters for control plants and upper case letters for aphid‐infested plants (*P* ≤ 0.05). Abbreviations: DM, dry matter; 4MSOB, 4‐methylsulfinylbutyl GLS; 4MTB, 4‐methylthiobutyl GLS; I3M, indole‐3‐yl methyl GLS; 4MO‐I3M, 4‐methoxy‐indole‐3‐yl‐methyl GLS [Colour figure can be viewed at http://wileyonlinelibrary.com]

Although the levels of most short‐ and long‐chain aliphatic GLS in undamaged and aphid‐damaged leaf tissues were comparable among plant types (Figure S3), levels of a major aliphatic GLS, 4‐methylsulfinylbutyl GLS (4MSOB), in *hds3* leaf tissues were significantly lower than the levels in WT and 35S:HDS (*hds3*) plants before and after aphid feeding (Figure 5a; one‐way ANOVA; *P*_(control)_ = 0.05, *P*_(aphid)_ = 0.01). Concomitantly, the levels of 4‐methylthiobutyl GLS (4MTB), a precursor of 4MSOB, were significantly higher in undamaged and aphid‐damaged leaf tissue of *hds3* plants than in leaf tissue of WT or 35S:HDS (*hds3*) plants regardless of aphid infestation (Figure 5b; one‐way ANOVA; *P*_(control)_ = 0.01, *P*_(aphid)_ = 0.01).

Although total levels of indole GLS in undamaged and aphid‐damaged plants did not differ among the three genotypes (Figure S4C; one‐way ANOVA; *P*_(undamaged)_ = 0.98, *P*_(aphid)_ = 0.12), the levels of indole‐3‐yl methyl (I3M) GLS in undamaged or aphid‐damaged leaf tissue of *hds3* plants were significantly lower than the levels in undamaged or aphid‐damaged tissue of WT or 35S:HDS (*hds3*) leaves (Figure 5c; one‐way ANOVA; *P*_(undamaged)_ = 0.01, *P*_(aphid)_ = 0.03). Furthermore, the levels of 4‐methoxy‐indole‐3‐yl‐methyl GLS (4MO‐I3M) in undamaged or aphid‐damaged *hds3* plants were significantly higher than the levels in undamaged or aphid‐damaged leaves of WT or of 35S:HDS (*hds3*) plants (Figure 5d; one‐way ANOVA; *P*_(undamaged)_ = 0.001, *P*_(aphid)_ = 0.001).

To investigate the effect of plant type and aphid infestation on the alteration of specific aliphatic and indolic GLS derivatives, we analysed these GLS levels against plant type (WT, *hds3*, and 35S:HDS (*hds3*) plants) and treatment conditions (undamaged and aphid damaged) by two‐way ANOVA. Plant type had a significant impact on the levels of all GLS derivatives (Table 1; two‐way ANOVA; *P*_(4MSOB)_ ≤ 0.001, *P*_(4MTB)_ ≤ 0.001, *P*_(I3M)_ ≤ 0.001, *P*_(4MO‐I3M)_ ≤ 0.001), whereas the effect of treatment was not significant (Table 1; two‐way ANOVA; *P*_(4MSOB)_ = 0.106, *P*_(4MTB)_ = 0.088, *P*_(I3M)_ = 0.670, *P*_(4MO‐I3M)_ = 0.763). Furthermore, the interaction of plant type and treatment was not significant (Table 1; two‐way ANOVA; *P*_(4MSOB)_ = 0.414, *P*_(4MTB)_ = 0.438, *P*_(I3M)_ = 0.401, *P*_(4MO‐I3M)_ = 0.598). Together, these results indicate that the alteration of 4MSOB, 4MTB, I3M, and 4MO‐I3M GLS levels is caused by the effect of the mutation in the *HDS* gene in *hds3* mutant plants.

**Table 1:** Summary of two‐way analysis of variance of the impact of plant type (wild type, *hds3* mutant and 35S:HDS in *hds3* background) and treatment (undamaged and aphid damaged) on the levels of specific aliphatic and indolic glucosinolates

Glucosinolates	Plant type		Treatment		Plant*treatment	
	*F* value	*P* value	*F* value	*P* value	*F* value	*P* value
4MSOB	10.124	**<0.001** ^***^	2.827	0.106	0.916	0.414
4MTB	23.577	**<0.001** ^***^	3.166	0.088	0.854	0.438
I3M	11.987	**<0.001** ^***^	0.186	0.670	0.950	0.401
4MO‐I3M	31.071	**<0.001** ^***^	0.093	0.763	0.525	0.598

*Note*. Significant effects are presented in bold. 4MSOB: 4‐methylsulfinylbutyl GLS; 4MTB: 4‐methylthiobutyl GLS; I3M: indole‐3‐yl methyl GLS; 4MO‐I3M: 4‐methoxy‐indole‐3‐yl‐methyl GLS

**Figure 6:**
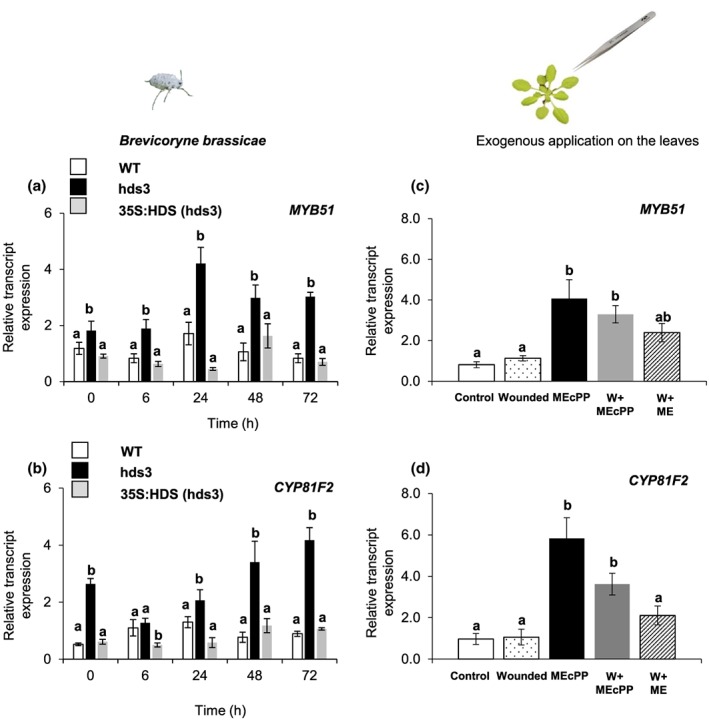
Relative transcript abundance of selected genes involved in indole glucosinolate metabolism, after *Brevicoryne brassicae* aphid feeding or after exogenous application of MEcPP. Relative transcript expression (means ± *SE*; *n* = 5) of (a) a regulator of indolic GLS (a) *MYB51* transcription factor and (b) *CYP81F2*, coding for a cytochrome P450 monooxygenase that catalyses the conversion of I3M into 4MO‐I3M for WT, *hds3*, and 35S:HDS (*hds3*) plants at designated time points of aphid infestation are presented. Transcript expression levels of (c) *MYB51* transcription factor and (d) CYP81F2 in wild type leaf tissues was determined at 2 hr after exogenous application of synthetic MEcPP or ME. Transcript expression levels after aphid infestation or after exogenous application of MEcPP are compared by one‐way ANOVA followed by Tukey's honestly significant difference (HSD) posthoc test. Different letters indicate significant differences among plant genotypes (*P* ≤ 0.05). Abbreviation: MEcPP, 2‐*C*‐methyl‐*D*‐erythritol‐2,4‐cyclodiphosphate; ME, methyl‐*D*‐erythritol; DM, dry matter [Colour figure can be viewed at http://wileyonlinelibrary.com]

The alteration of 4MSOB, 4MTB, I3M, and 4MO‐I3M GLS levels as observed in *hds3* plants (Figure 5) was very similar to the change in GLS profile when overexpressing *MYB51* transcription factor (TF), a major transcriptional regulator of indole GLS, in Arabidopsis (Frerigmann & Gigolashvili, 2014; Gigolashvili, Yatusevich, Berger, Muller, & Flugge, 2007). We examined whether *MYB51* was involved in a specific change in GLS profile in *hds3* plants by quantifying the kinetics of the relative transcript levels of *MYB51* in WT, *hds3*, and 35S:HDS (*hds3*) plants before and after *B. brassicae* feeding. Additionally, the relative transcript levels of the gene coding for cytochrome P450 monooxygenase family 81 subfamily F2 (*CYP81F2*), a gene catalysing the conversion of I3M into 4MO‐I3M (Pfalz, Vogel, & Kroymann, 2009), was also quantified. The relative transcript levels of *MYB51* and *CYP81F2* were significantly higher in undamaged *hds3* plants compared with undamaged WT or 35S:HDS (*hds3*) plants (Figure 6a,b; one‐way ANOVA; *P*_(MYB51)_ = 0.04, *P*_(CYP81F2)_ = 0.01). Furthermore, relative transcript levels of *MYB51* and *CYP81F2* in *hds3* plants were also significantly higher at the later time points (24 or 48 hr) after *B. brassicae* aphid feeding compared with the expression at the constitutive level (Figure 6a,b; one‐way ANOVA; *P*_(MYB51‐24h)_ = 0.01; *P*_(CYP81F2‐48h)_ = 0.01). Unlike for *hds3* plants, transcript levels of *MYB51* and *CYP81F2* did not increase upon *B. brassicae* feeding in WT or 35S:HDS (*hds3*) plants (Figure 6a,b). Our results show positive correlations of high relative expression of *MYB51* and *CYP81F2* with a change in GLS profile in *hds3* plants. Based on these results, we hypothesize that MEcPP could be a direct regulator of *MYB51* and *CYP81F2*, leading to an alteration of specific GLS in *hds3* plants.

### 3.4 MEcPP regulates MYB51 and CYP81F2 expression in Arabidopsis

To explore whether MEcPP exerts direct control over the expression of *MYB51* and *CYP81F2*, we analysed relative transcript levels of *MYB51* and *CYP81F2* at 2 hr after exogenous application of synthetic MEcPP onto WT leaves. Besides a high accumulation of MEcPP, *hds3* plants also accumulate high levels of MEcPP‐related metabolites including MEP and ME (Figure 4). Therefore, we also tested whether MEcPP‐related metabolites are active metabolites that regulate transcript expression of *MYB51* and *CYP81F2* by exogenous application of synthetic ME on WT leaves. As WT leaves were wounded by fine tweezers prior to an exogenous application of MEcPP or ME to facilitate their entrance into cells (Section 2), we also quantified relative transcript expression of *MYB51* and *CYP81F2* in wounded leaves to assess the effect of wound‐induced expression of these genes.

Although the expression of *MYB51* and *CYP81F2* was not induced 2 hr after mechanical wounding (Figure 6c,d; one‐way ANOVA; *P*_(MYB51‐wounded)_ = 0.99, *P*_(CYP81F2‐wounded)_ = 0.90), the relative expression of *MYB51* and *CYP81F2* was significantly increased (~2‐ to 3‐fold) in MEcPP and wounded + MEcPP (W + MEcPP) treatments (Figure 6c,d; one‐way ANOVA; *P*_(MYB51‐MEcPP)_ = 0.01, *P*_(MYB51‐W + MEcPP)_ = 0.03, *P*_(CYP81F2‐MEcPP)_ = 0.01, *P*_(CYP81F2‐W + MEcPP)_ = 0.05). The expression of *MYB51* and *CYP81F2* was not significantly increased in wounded + ME (W + ME) treatment compared with an untreated control leaf (Figure 6c,d; one‐way ANOVA; *P*_(MYB51‐W + ME)_ = 0.23, *P*_(CYP81F2‐W + ME)_ = 0.76). In addition, the expression of *ICS1* and *PR1* was also significantly increased in response to MEcPP and W + MEcPP treatments (Figure S5; one‐way ANOVA; *P*_(ICS1‐MEcPP)_ = 0.05, *P*_(ICS1‐W + MEcPP)_ = 0.02; *P*_(PR1‐MEcPP)_ = 0.01; *P*_(PR1‐W + MEcPP)_ = 0.05). Together, our results indicate that MEcPP, but not the free alcohol form ME, exerts direct control over the expression of *MYB51* and *CYP81F2*, which thereafter contributes to an increase in 4MO‐I3M GLS in *hds3* plants.

### 3.5 MEcPP impacts the expression of other genes mediating plant resistance to aphids

**Figure 7:**
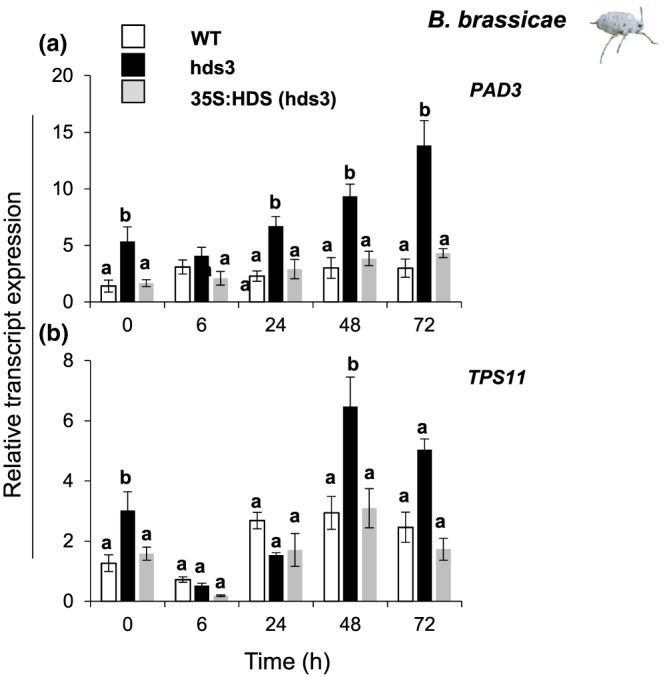
Differential transcript expression of *PAD3* and *TPS11* in *hds3* mutant plants. Relative transcript levels (means ± *SE*; *n* = 5) of (a) camalexin biosynthetic gene (*PAD3*), (b) a key gene in trehalose metabolisms (*TPS11*). Transcript expression were determined in WT, *hds3*, and 35S:HDS (*hds3*) leaves. They were compared among plant types at each time point by one‐way ANOVA followed by Tukey's honestly significant difference (HSD) posthoc test. Different letters indicate significant differences among plant genotypes (*P* ≤ 0.05). Abbreviations: DM, dry matter; *PAD3*, *PHYTOALEXIN DEFICIENT3*; *TPS11*, *TREHALOSE‐6‐PHOSPHATE SYNTHASE11* [Colour figure can be viewed at http://wileyonlinelibrary.com]

Although GLS are important defence metabolites against herbivores including aphids, it has been widely reported that some aphid species such as *B. brassicae* are able to sequester GLS (Kazana et al., 2007; Kos et al., 2012). Recent studies of aphid resistance in Arabidopsis showed that other metabolites including the indole alkaloid, camalexin, and an alpha‐linked glucose disaccharide (trehalose) could function in the defence against the aphids *B. brassicae* and *M. persicae* (Kuśnierczyk et al., 2008; Mewis, Khan, Glawischnig, Schreiner, & Ulrichs, 2012; Singh et al., 2011). As *hds3* plants were resistant to *B. brassicae* aphids, we examined whether the accumulation of MEcPP in *hds3* plants affected camalexin and trehalose metabolism. The relative expression of selected genes in camalexin biosynthesis and trehalose metabolism were assessed in undamaged and aphid‐damaged leaf tissues of WT, *hds3*, and 35S:HDS (*hds3*) plants. Relative expression of *PHYTOALEXIN DEFICIENT3 (PAD3*) and *TREHALOSE‐6‐PHOSPHATE SYNTHASE11 (TPS11)*, a key gene in camalexin biosynthesis and trehalose metabolism respectively, was significantly higher in undamaged *hds3* plants than in undamaged WT or 35S:HDS (*hds3*) plants (Figure 7a,b; one‐way ANOVA; *P*_(PAD3‐undamaged)_ = 0.01; *P*_(TPS11‐undamaged)_ = 0.03). The relative expression of *PAD3* and *TPS11* in leaf tissues of WT or 35S:HDS (*hds3*) plants hardly changed upon aphid feeding. However, relative expression of *PAD3* and *TPS11* in *hds3* plants significantly increased from the levels in undamaged *hds3* leaves after 48 and 72 hr of *B. brassicae* feeding (Figure 7a,b; one‐way ANOVA; *P*_(PAD3‐48h)_ = 0.05, *P*_(PAD3‐72h)_ = 0.001, *P*_(TPS11‐48h)_ = 0.003, *P*_(TPS11‐72h)_ = 0.04). An increase in transcript level of *PAD3* and *TPS11* at the later time points after aphid feeding, as observed in *hds3* plants, coincided with an increased expression of *MYB51* and *CYP81F2* at the same time points (Figure 6a,b). Overall, these results support the assumption that MEcPP functions as a positive regulator of diverse metabolic pathways which in turn confer resistance to the cabbage aphid *B. brassicae* in Arabidopsis.

## 4 DISCUSSION

### 4.1 High SA level in *hds3* plants does not interfere with the JA‐signalling pathway after caterpillar feeding or with caterpillar resistance

That high MEcPP levels are associated with an increased SA level that has been previously described for *hds3* and *ceh1* mutants (Gil et al., 2005; González‐Cabanelas et al., 2015; Xiao et al., 2012). As antagonistic interactions between the SA‐ and JA‐signalling pathways are known to suppress plant resistance to caterpillars such as the cabbage looper (*Trichoplusia ni*) (Cui et al., 2002), we investigated the impact of the high basal level of SA in *hds3* plants on JA‐induced plant resistance to *P. brassicae* caterpillars. Resistance to *P. brassicae* is mediated by JA‐signal transduction (Reymond et al., 2004). Here, we found that *hds3* and WT plants are equally resistant to *P. brassicae* caterpillars (Figure 3a). Furthermore, the transcript expression in *hds3* plants of genes involved in JA biosynthesis (*AOS*), JA response (*VSP2*), and a JA‐inducible gene (*ERF1*) was equally induced in response to *P. brassicae* caterpillar feeding as in WT plants (Figure 3d–f). These results indicate that an increased expression of genes in the SA‐signalling pathway induced through an elevation of the MEcPP level does not interfere with the caterpillar‐induced expression of genes in the JA‐signalling pathway or with resistance to *P. brassicae* caterpillars. Moreover, our results are consistent with previous studies that a high SA level did neither attenuate transcript expression of genes in the JA‐signalling pathway nor the accumulation of JA in plants with high MEcPP levels, that is, *hds3* and *ceh1* mutant plants (González‐Cabanelas et al., 2015; Lemos et al., 2016). It is noteworthy that *ceh1* mutant plants used by Lemos et al. (2016) are the result of a point mutation in exon 19 at leucine^703^ to phenylalanine (Xiao et al., 2012), whereas the *hds3* mutant plants are generated through a point mutation at glycine^696^ to aspartic acid in the same exon (Gil et al., 2005). Furthermore, *ceh1* mutant plants have originally been screened to identify novel genes that control the transcript expression of *hydroperoxide lyase* (*HPL*), encoding an enzyme in the green‐leaf volatile biosynthesis pathway (Lemos et al., 2016; Xiao et al., 2012). Although *hds3* plants share various phenotypes with *ceh1* plants, the basal expression of *HPL* in *hds3* plants was comparable with the expression in WT plants (Figure S6). As the HPL pathway is known to compete for substrate with the JA‐biosynthesis pathway (Chehab et al., 2006), it is intriguing to further investigate the impact of MEcPP on the different expression levels of *HPL* on green‐leaf volatile production in *ceh1* and *hds3* mutant plants as well as the possible impacts of these changes on indirect plant defence (Van Poecke & Dicke, 2004) to herbivory.

Although our results show that enhanced SA signalling in *hds3* mutant plants did not suppress JA signalling (Figure 3), we observed a negative impact of JA‐signalling on the SA‐signalling pathway. We found that the reduction in *ICS1* expression at 6 hr of caterpillar feeding coincided with the induction of *AOS* transcript expression at the same time point in *hds3* plants (Figure 3). Even though SA signalling has been frequently reported to suppress JA signalling, several studies have reported antagonistic effects of JA‐signalling on the SA‐signalling pathway (Kachroo, Shanklin, Shan, Whittle, & Klessing, 2001; Salzman et al., 2005; Thaler, Fidantsef, & Bostock, 2002; Thaler, Humphrey, & Whiteman, 2012).

### 4.2 Alteration of MEcPP levels upon *B. brassicae* aphid infestation

Isoprenoid metabolites have important roles in various biological processes ranging from photosynthesis to responses to environmental stresses. Hence, it is important for plants to develop sophisticated mechanisms for synthesis and reallocation of isoprenoid precursors to different tissues under different circumstances in plant life cycles (Flores‐Peréz, Sauret‐Gueto, Gas, Jarvis, & Rodriguez‐Concepcion, 2008; Li & Sharkey, 2013; Wright et al., 2014). It has been hypothesized that plants possess an isolated pool of MEcPP that can be exported from the plastid during an increased metabolite flux through the MEP pathway to protect metabolic flux shifting in the MEP pathway (González‐Cabanelas et al., 2015; Wright et al., 2014). It is interesting that we found a significant accumulation of only MEcPP‐ and MEcPP‐related metabolites, whereas other metabolites in the MEP pathway were unaffected after aphid feeding on WT and *hds3* plants (Figure 4). Furthermore, an increased expression of *HDS* at later time points (24, 48, and 72 hr) since the initiation of aphid feeding in WT plants (Figure 2B) suggested an increased activity in the MEP pathway. Together, our findings suggest that aphid infestation increases metabolic flux in the MEP pathway, which activates an efflux of a secondary pool of MEcPP from the plastid, triggering transcriptional reprogramming in the nucleus. Hence, future research to quantify metabolic flux of the MEP pathway upon aphid infestation and the identification of MEcPP efflux protein(s) will provide essential information for metabolic engineering of MEcPP to enhance plant resistance to the *B. brassicae* aphids.

### 4.3 MEcPP and plant resistance against specialist aphids

Specialist herbivores are highly tolerant to host‐plant defence mechanisms, for example, due to the ability to detoxify and/or sequester defence metabolites of their host plant (Schoonhoven, van Loon, & Dicke, 2005). *B. brassicae* is a specialist herbivore feeding on brassicaceous plants, including Arabidopsis. These aphids are known for their ability to sequester GLS and utilize sequestered GLS in defence against their predators (Schoonhoven et al., 2005). Unlike generalist aphids, many studies show that SA signalling does not have a significant role in mediating plant defences against *B. brassicae* aphids. SA‐deficient Arabidopsis mutant plants (*sid2‐1*) exhibited a similar level of resistance to *B. brassicae* as WT plants (Onkokesung et al., 2016). Hence, an elevation of the expression of marker genes in SA‐biosynthesis (*ICS1*) and SA‐response (*PR1*) pathways of *hds3* plants (Figure 2) cannot fully explain an enhanced resistance against *B. brassicae* aphids in the mutant plants. In contrast, a significant increase in gene transcription in the SA‐signalling pathway may protect *hds3* plants from pathogens. Indeed, *hds3* plants are more resistant to a biotrophic pathogen, *Hyaloperonospora parasitica*, than WT plants (Gil et al., 2005).

Indole GLS, especially 4MO‐I3M GLS, provide effective defence against generalist aphids such as *M. persicae* (Kim & Jander, 2007). Furthermore, Kuśnierczyk et al. (2008) reported that 4MO‐I3M GLS levels were significantly increased 48 hr after *B. brassicae* feeding on Arabidopsis ecotype Landsberg *erecta*. Here, we found that 4MO‐I3M GLS levels did not increase after *B. brassicae* feeding on Arabidopsis ecotype Col‐0 plants (Figure 5d). Our results are consistent with a previous study that showed no significant induction of 4MO‐I3M GLS after *B. brassicae* aphid feeding on ecotype Col‐0 (Onkokesung et al., 2014). In contrast, *hds3* plants contain high levels of 4MO‐I3M before and after aphid infestation (Figure 5d). Because a high 4MO‐I3M level correlates with enhanced resistance against *B. brassicae* aphids in *hds3* plants, future studies to experimentally prove that 4MO‐I3M confers resistance to this aphid species will be relevant.

The phytoalexin camalexin is a metabolite providing effective defence against cabbage aphids. For instance, camalexin‐deficient mutant plants (*pad3‐1*) were more susceptible to *B. brassicae* aphids than WT plants (Kuśnierczyk et al., 2008). Indeed, here, we found a positive correlation between a high relative expression of *PAD3* and enhanced resistance to *B. brassicae* aphids in *hds3* mutant plants (Figures 7a and 2). Future research to quantify camalexin concentrations in phloem sap of WT and *hds3* plants before and after *B. brassicae* feeding will provide further information on the regulatory functions of MEcPP on camalexin biosynthesis and plant resistance against *B. brassicae*.

In recent years, T6P has been reported as a signalling molecule mediating plant responses to drought stress, generalist aphid feeding, and plant productivity (Griffiths et al., 2016; Singh et al., 2011). Arabidopsis mutants silenced in the expression of *TPS11*, an important gene in T6P metabolism, were more susceptible to *M. persicae* feeding than WT plants (Singh et al., 2011). Furthermore, the transcript expression of Sl*TPS11*, a homologue of Arabidopsis *TPS11* in tomato (*Solanum lycopersicum*), was upregulated after *M. persicae* feeding on tomato plants (Singh & Shah, 2012). These studies suggest an involvement of the trehalose metabolic pathway in the modulation of plant resistance against this aphid species. Our data show high relative transcript levels of *TPS11* specifically in *hds3* plants after 48 and 72 hr of *B. brassicae* feeding (Figure 7b). Previous studies proposed that *TPS11* was involved in the reallocation of sucrose to starch (Singh et al., 2011), as a mechanism of plants to reduce the nutritional value of the phloem sap to interfere with aphid feeding. In fact, starch‐deficient Arabidopsis mutants (*pgm1*) were more susceptible to *M. persicae* than WT plants (Singh et al., 2011). Hence, future studies to quantify trehalose, sucrose, and starch levels in *hds3* plants will provide significant information to link MEcPP and sugar signalling to the mechanisms of plant resistance to aphid infestation.

Overall, our data provide important evidence to support a novel function of MEcPP as a regulator at the transcriptional level of various molecular mechanisms underlying plant resistance to the aphid *B. brassicae* in Arabidopsis. The effect of MEcPP on enhancing the SA‐signalling pathway without interfering with the JA‐signalling pathway provides evidence of a regulatory network that works upstream of plant hormonal signalling networks and their interactions in Arabidopsis. Further studies on MEcPP transporters and the prospective MEcPP receptors will provide important information for metabolically engineering MEcPP to enhanced host plant resistance against *B. brassicae* aphids.

## AUTHOR CONTRIBUTIONS

N.O. and M.D. designed the research. N.O., M.R., L.P.W., and M.A.P. performed the research and analysed the data. N.O. and M.D. wrote the paper with input from M.R., M.A.P., and J.G.

## Supporting information


**Table S1** Specific primers used for quantitative real‐time PCR.
**Figure S1. Time course of relative transcript expression of *HDS* in response to feeding by** ***Pieris brassicae*** **caterpillars on (A) WT and (B) 35S:HDS (*hds3)* plants.** The graphs represent average expression ± SE (*n* = 5) at each time point. The expression was compared among time point of each plant type by one‐way ANOVA followed by Tukey's honestly significant difference (HSD) posthoc test). Different letters above bars indicate significant differences among time points (*P* ≤ 0.05).
**Figure S2. Accumulation of an intermediate metabolite and final metabolites of the MEP pathway in undamaged and aphid‐damaged leaf tissue of WT, *hds3*, and 35S:HDS (*hds3*) plants.** The effects of a high accumulation of MEcPP on other metabolites in MEP pathway were determined by quantification via HPLC/MS of (A) the intermediate metabolite 1‐deoxy‐D‐xylulose 5‐phosphate DXP and (B) the final metabolites isopentenyl diphosphate (IPP) and dimethylallyl diphosphate (DMAPP) in undamaged (control) and aphid‐damaged tissue from WT, *hds3* and 35S:HDS (h*ds3*) plants. Values represent average ± SE (*n* = 5) of DXP and IPP+DMAPP levels in leaf tissue of each plant type. Data were compared within the genotype by Student's *t*‐test, different letters indicate significant difference (*P* ≤ 0.05).
**Figure S3. Accumulation of aliphatic glucosinolates in undamaged and aphid damaged leaf tissue of WT, *hds3*, or 35S:HDS (*hds3*) plants**. The aliphatic glucosinolates were quantified by HPLC/MS analysis of leaf tissue of WT, *hds3* and 35S:HDS (h*ds3*) plants. Values represent means ± SE (*n* = 5) for undamaged (A) and aphid‐infested (7d) (B) plants. Data were compared among WT, *hds3* and 35S:HDS (*hds3*) plants by one‐way ANOVA followed by Tukey's honestly significant difference (HSD) posthoc test. Different letters indicate significant differences between plant genotypes (*P* ≤ 0.05).
Abbreviations: 3MSOP: 3‐methylsulphinylpropyl glucosinolate; 4MTB: 4‐methylthiobutyl glucosinolate; 4MSOB: 4‐methylsulphinylbutyl glucosinolate; 7MSOH: 7‐methylsulphinylheptyl glucosinolate; 8MSOO: 8‐methylsulphinyloctyl glucosinolate.
**Figure S4. Accumulation of aliphatic, indolic and total glucosinolate levels in undamaged and aphid‐damaged leaf tissue of WT, *hds3*, or 35S:HDS (*hds3*) plants.** Impact of a high accumulation of MEcPP on glucosinolate accumulation in response to cabbage aphid feeding. Aliphatic glucosinolates and indolic glucosinolates were quantified by HPLC/MS for undamaged or aphid‐damaged leaf tissue of WT, *hds3* and 35S:HDS (h*ds3*) plants. The sum of (A) aliphatic glucosinolates, (B) indolic glucosinolates and (C) the total levels of (aliphatic+indolic) glucosinolates are presented. The data were compared among WT, *hds3* and 35S:HDS (*hds3*) plants by one‐way ANOVA followed by Tukey's honestly significant difference (HSD) posthoc test. Different letters indicate significant differences within each plant genotype (*P* ≤ 0.05).
**Figure S5. Relative transcript expression of a marker gene of SA biosynthesis and a marker gene of SA response in WT leaves in response to an exogenous application of synthetic MEcPP or ME.** Synthetic MEcPP or ME was exogenously applied on a fully expanded leaf of wild type plants to determine the effects of MEcPP on the SA signalling pathway. The relative transcript expression of SA biosynthetic gene (*ICS1*) and a marker gene of SA responsive gene (*PR1*) was quantified at 2h after exogenous application of MEcPP or ME. The average transcript expression ± SE (*n* = 4) is presented. Data were compared among treatments by one‐way ANOVA followed by Tukey's honestly significant difference (HSD) posthoc test. Different letters indicate significant differences among treatments (*P* ≤ 0.05).
**Figure S6. Kinetics of the relative transcript expression of *hydroperoxide lyase* (*HPL*) in response to feeding by the aphid** ***B. brassicae*** **in leaf tissue of WT, *hds3*, or 35S:HDS (*hds3*) plants**. Average transcript expression levels ± SE (*n* = 5) are presented for different time points Data were compared among plant genotypes at each time point by one‐way ANOVA followed by Tukey's honestly significant difference (HSD) posthoc test. Different letters indicate significant differences among plant genotype (*P* ≤ 0.05)
