# Supplementary material for: The plastidial metabolite 2‐C‐methyl‐D‐erythritol‐2,4‐cyclodiphosphate modulates defence responses against aphids
Source: Plant Cell Environ. 2019 Mar 8;42(7):2309–23. doi: 10.1111/pce.13538 (PMC6850158; doi:10.1111/pce.13538)
Supplement: Supplementary file 1 — Table S1 Specific primers used for quantitative real‐time PCR. Figure S1. Time course of relative transcript expression of HDS in response to feeding by Pieris brassicae caterpillars on (A) WT and (B) 35S:HDS (hds3) plants. The graphs represent average expression ± SE (n = 5) at each time point. The expression was compared among time point of each plant type by one‐way ANOVA followed by Tukey's honestly significant difference (HSD) posthoc test). Different letters above bars indicate significant differences among time points (P ≤ 0.05). Figure S2. Accumulation of an intermediate metabolite and final metabolites of the MEP pathway in undamaged and aphid‐damaged leaf tissue of WT, hds3, and 35S:HDS (hds3) plants. The effects of a high accumulation of MEcPP on other metabolites in MEP pathway were determined by quantification via HPLC/MS of (A) the intermediate metabolite 1‐deoxy‐D‐xylulose 5‐phosphate DXP and (B) the final metabolites isopentenyl diphosphate (IPP) and dimethylallyl diphosphate (DMAPP) in undamaged (control) and aphid‐damaged tissue from WT, hds3 and 35S:HDS (hds3) plants. Values represent average ± SE (n = 5) of DXP and IPP+DMAPP levels in leaf tissue of each plant type. Data were compared within the genotype by Student's t‐test, different letters indicate significant difference (P ≤ 0.05). Figure S3. Accumulation of aliphatic glucosinolates in undamaged and aphid damaged leaf tissue of WT, hds3, or 35S:HDS (hds3) plants. The aliphatic glucosinolates were quantified by HPLC/MS analysis of leaf tissue of WT, hds3 and 35S:HDS (hds3) plants. Values represent means ± SE (n = 5) for undamaged (A) and aphid‐infested (7d) (B) plants. Data were compared among WT, hds3 and 35S:HDS (hds3) plants by one‐way ANOVA followed by Tukey's honestly significant difference (HSD) posthoc test. Different letters indicate significant differences between plant genotypes (P ≤ 0.05). Abbreviations: 3MSOP: 3‐methylsulphinylpropyl glucosinolate; 4MTB: 4‐methylthiobutyl glucosino [file PCE-42-2309-s001.pdf]

Supporting Information

Article title: *The 2-C-methyl-D-erythritol-2,4-cyclodiphosphate metabolite modulates defense responses against aphid in Arabidopsis*

Authors: Nawaporn Onkokesung[1†], Michael Reichelt[2], Louwrance P. Wright[2], Michael A. Phillips[3], Jonathan Gershenzon,[2] and Marcel Dicke[1*]

The following Supporting Information is available for this article:

Table S1 Specific primers used for quantitative real-time PCR.

Gene	Sequence (5´→3´)
**HDS-Forward**	CAGAATGCGTAACACTAAGAC
**HDS-Reverse**	GAGAACCACCTACATATCCG
**MYB51-Forward**	CTACAAGTGTTTCCGTTGACTCTGAA
**MYB51-Reverse**	ACGAAATTATCGCAGTACATTAGAGGA
**CYP81F2-Forward**	TATTGTCCGCATGGTCACAGG
**CYB81F2-Reverse**	CCACTGTTGTCATTGATGTCCG
**WRKY70-Forward**	ACCCGTTAAGGGTAAAAGAGGA
**WRKY70-Reverse**	CTTGGGTTCGAGCTCAACCT
**AOS-Forward**	TCCACCCAAAAACCGTACGA
**AOS-Reverse**	TGAAGAACTCTTCAGCTCCTTG
**HPL-Forward**	GCTGAGAACGGTTGGAAAAC
**HPL-Reverse**	TCCGGCGATTAAGAGAGAAG
**ICS1-Forward**	CACTAGATTCTCCCGCAAGAAG
**ICS1-Reverse**	TGGTCAATTGGAACCTGTAACC
**VSP2-Forward**	TCAGTGACCGTTGGAAGTTGTG
**VSP2-Reverse**	GTTCGAACCATTAGGCTTCAATATG
**PR1-Forward**	CACTACACTCAAGTTGTTTGGA
**PR1-Reverse**	TAGTATGGCTTCTCGTTCACAT
**PAD3-Forward**	CTGATCAGAAACCCAAGAGTGA
**PAD3-Reverse**	GTTTTCGCAGGAACATCGTAG
**PAD4-Forward**	AACAGAGATATAAAGACTGGCGGGC
**PAD4-Reverse**	ACACACTCCTCAGGCACTTTAACTC
**TPS11-Forward**	TGGTTCTCGTGCAGATTAC
**TPS11-Reverse**	TCCGAGATCGCGTAATAAG
**EF-Forward**	TGAGCACGCTCTTCTTGCTTTCA
**EF-Reverse**	GGTGGTGGCATCCATCTTGTTACA

Figure S1. Time course of relative transcript expression of *HDS* in response to feeding by *Pieris brassicae* caterpillars on (A) WT and (B) 35S:HDS (*hds3)* plants. The graphs represent average expression ± SE (n = 5) at each time point. The expression was compared among time point of each plant type by one-way ANOVA followed by Tukey's honestly significant difference (HSD) posthoc test). Different letters above bars indicate significant differences among time points ($P \leq 0.05$).

Figure S2. Accumulation of an intermediate metabolite and final metabolites of the MEP pathway in undamaged and aphid-damaged leaf tissue of WT, *hds3,* and 35S:HDS (*hds3*) plants. The effects of a high accumulation of MEcPP on other metabolites in MEP pathway were determined by quantification via HPLC/MS of (A) the intermediate metabolite 1-deoxy-D-xylulose 5-phosphate DXP and (B) the final metabolites isopentenyl diphosphate (IPP) and dimethylallyl diphosphate (DMAPP) in undamaged (control) and aphid-damaged tissue from WT, *hds3* and 35S:HDS (h*ds3*) plants. Values represent average ± SE (n = 5) of DXP and IPP+DMAPP levels in leaf tissue of each plant type. Data were compared within the genotype by Student's *t*-test, different letters indicate significant difference ($P \leq 0.05$).

Figure S3. Accumulation of aliphatic glucosinolates in undamaged and aphid damaged leaf tissue of WT, *hds3*, or 35S:HDS(*hds3*) plants. The aliphatic glucosinolates were quantified by HPLC/MS analysis of leaf tissue of WT, *hds3* and 35S:HDS (h*ds3*) plants. Values represent means ± SE (n = 5) for undamaged (A) and aphid-infested (7d) (B) plants. Data were compared among WT, *hds3* and 35S:HDS (*hds3*) plants by one-way ANOVA followed by Tukey's honestly significant difference (HSD) posthoc test. Different letters indicate significant differences between plant genotypes ($P \leq 0.05$).

Abbreviations: 3MSOP: 3-methylsulphinylpropyl glucosinolate; 4MTB: 4-methylthiobutyl glucosinolate; 4MSOB: 4-methylsulphinylbutyl glucosinolate; 7MSOH: 7-methylsulphinylheptyl glucosinolate; 8MSOO: 8-methylsulphinyloctyl glucosinolate.

Figure S4. Accumulation of aliphatic, indolic and total glucosinolate levels in undamaged and aphid-damaged leaf tissue of WT, *hds3,* or 35S:HDS(*hds3*) plants. Impact of a high

accumulation of MEcPP on glucosinolate accumulation in response to cabbage aphid feeding. Aliphatic glucosinolates and indolic glucosinolates were quantified by HPLC/MS for undamaged or aphid-damaged leaf tissue of WT, *hds3* and 35S:HDS (h*ds3*) plants. The sum of (A) aliphatic glucosinolates, (B) indolic glucosinolates and (C) the total levels of (aliphatic+indolic) glucosinolates are presented. The data were compared among WT, *hds3* and 35S:HDS (*hds3*) plants by one-way ANOVA followed by Tukey's honestly significant difference (HSD) posthoc test. Different letters indicate significant differences within each plant genotype ($P \leq 0.05$).

Figure S5. Relative transcript expression of a marker gene of SA biosynthesis and a marker gene of SA response in WT leaves in response to an exogenous application of synthetic MEcPP or ME. Synthetic MEcPP or ME was exogenously applied on a fully expanded leaf of wild type plants to determine the effects of MEcPP on the SA signaling pathway. The relative transcript expression of SA biosynthetic gene (*ICS1*) and a marker gene of SA responsive gene (*PR1*) was quantified at 2h after exogenous application of MEcPP or ME. The average transcript expression ± SE (n = 4) is presented. Data were compared among treatments by one-way ANOVA followed by Tukey's honestly significant difference (HSD) posthoc test. Different letters indicate significant differences among treatments ($P \leq 0.05$).

Figure S6. Kinetics of the relative transcript expression of *hydroperoxide lyase* (*HPL*) in response to feeding by the aphid *B. brassicae* in leaf tissue of WT, *hds3*, or 35S:HDS(*hds3*) plants. Average transcript expression levels ± SE (n = 5) are presented for different time points Data were compared among plant genotypes at each time point by one-way ANOVA followed by Tukey's honestly significant difference (HSD) posthoc test. Different letters indicate significant differences among plant genotype ($P \leq 0.05$)

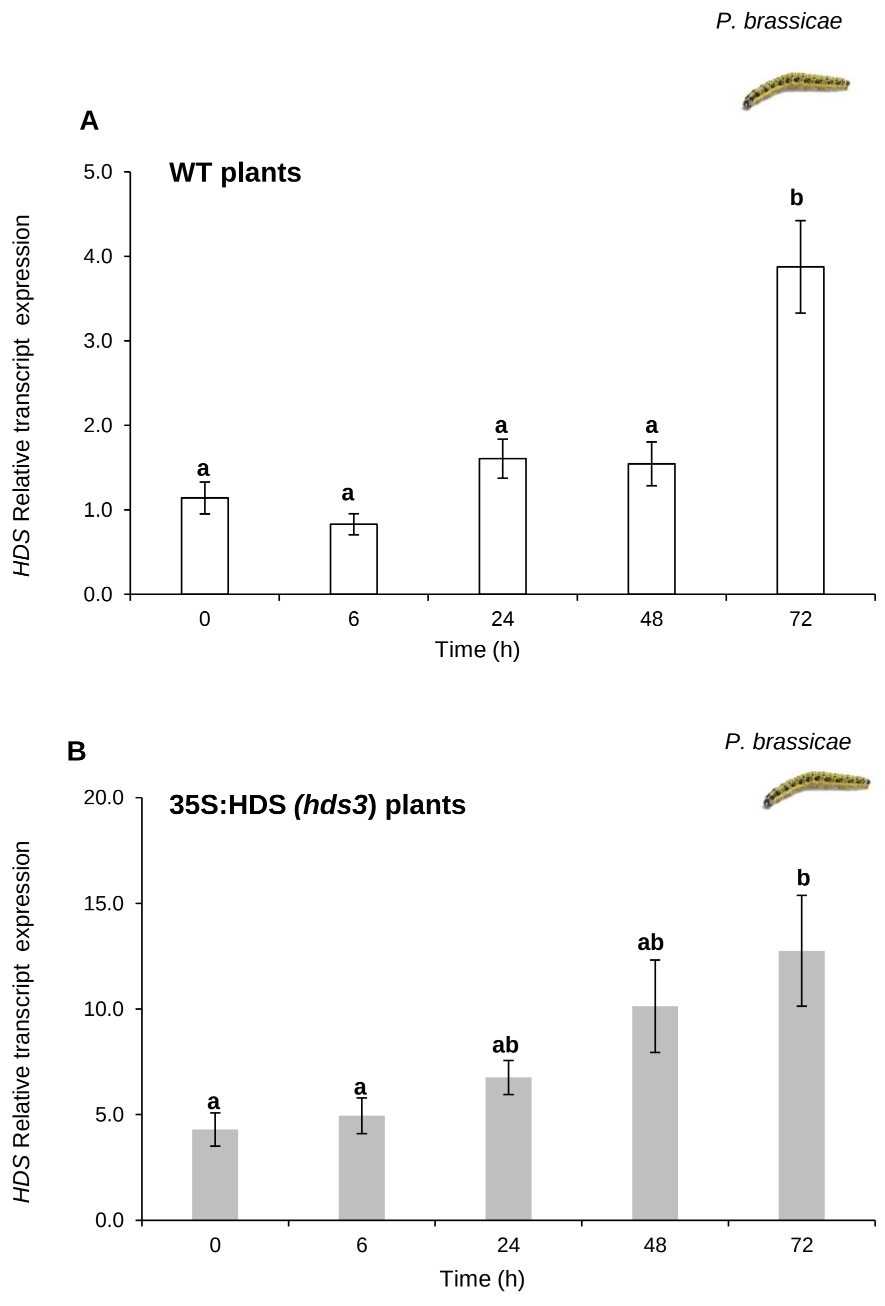
P. brassicae
A
WT plants
HDS Relative transcript expression
5.0
4.0
3.0
2.0
1.0
0.0
a
a
a
a
b
0
6
24
48
72
Time (h)
P. brassicae
B
35S:HDS (hds3) plants
HDS Relative transcript expression
20.0
15.0
10.0
5.0
0.0
a
a
ab
ab
b
0
6
24
48
72
Time (h)

Figure S1

Brevicoryne brassicae 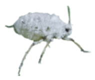

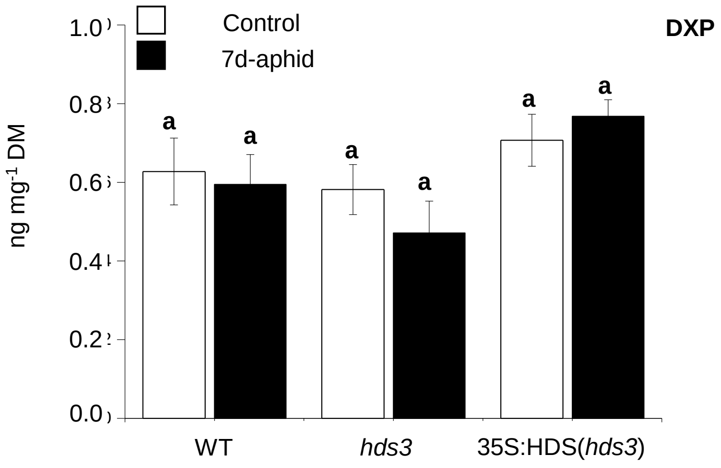

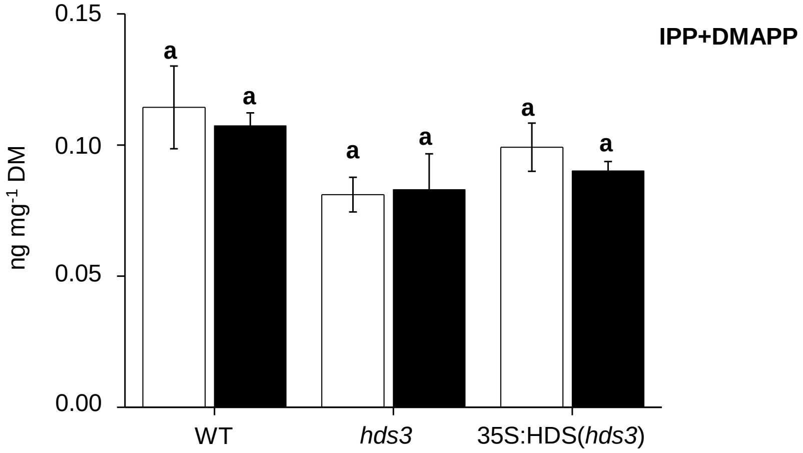

Figure S2

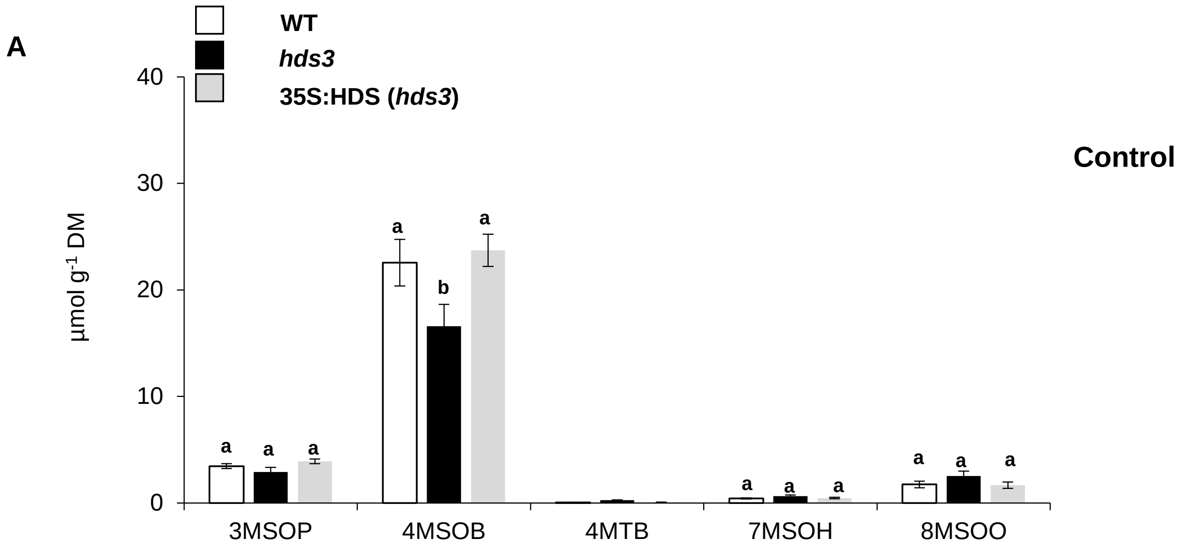

A
WT
hds3
35S:HDS (hds3)
Control
µmol g-1 DM
40
30
20
10
0
a
a
b
a
a
a
a
a
a
a
a
a
a
a
3MSOP
4MSOB
4MTB
7MSOH
8MSOO

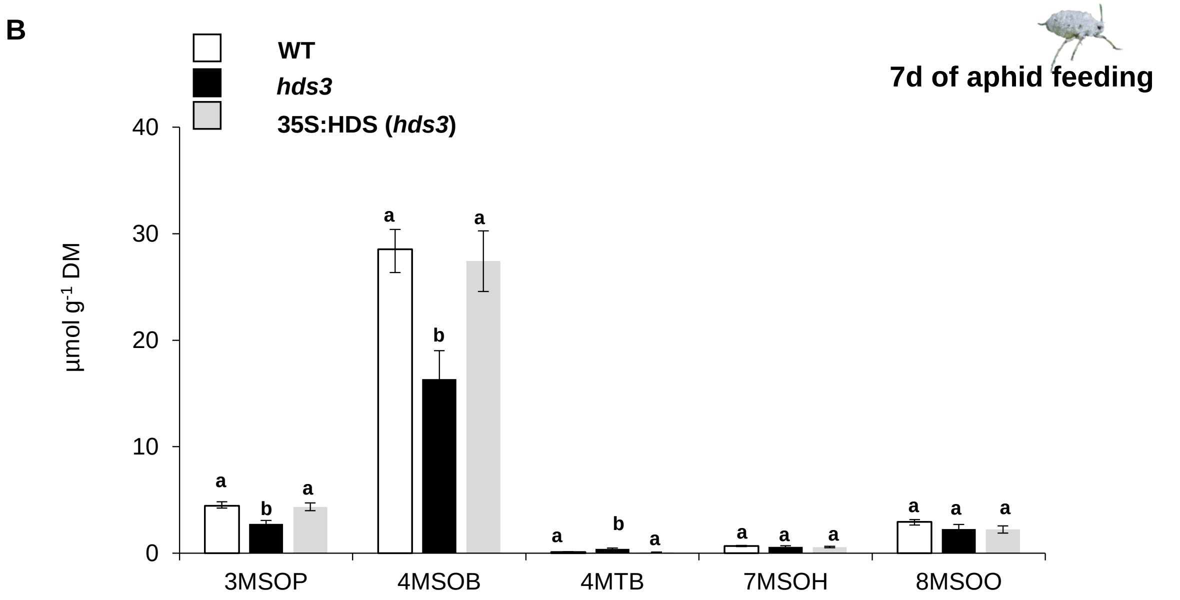

B
WT
hds3
35S:HDS (hds3)
7d of aphid feeding
µmol g-1 DM
40
30
20
10
0
a
a
b
a
b
a
a
b
a
a
a
a
a
a
a
3MSOP
4MSOB
4MTB
7MSOH
8MSOO

Figure S3

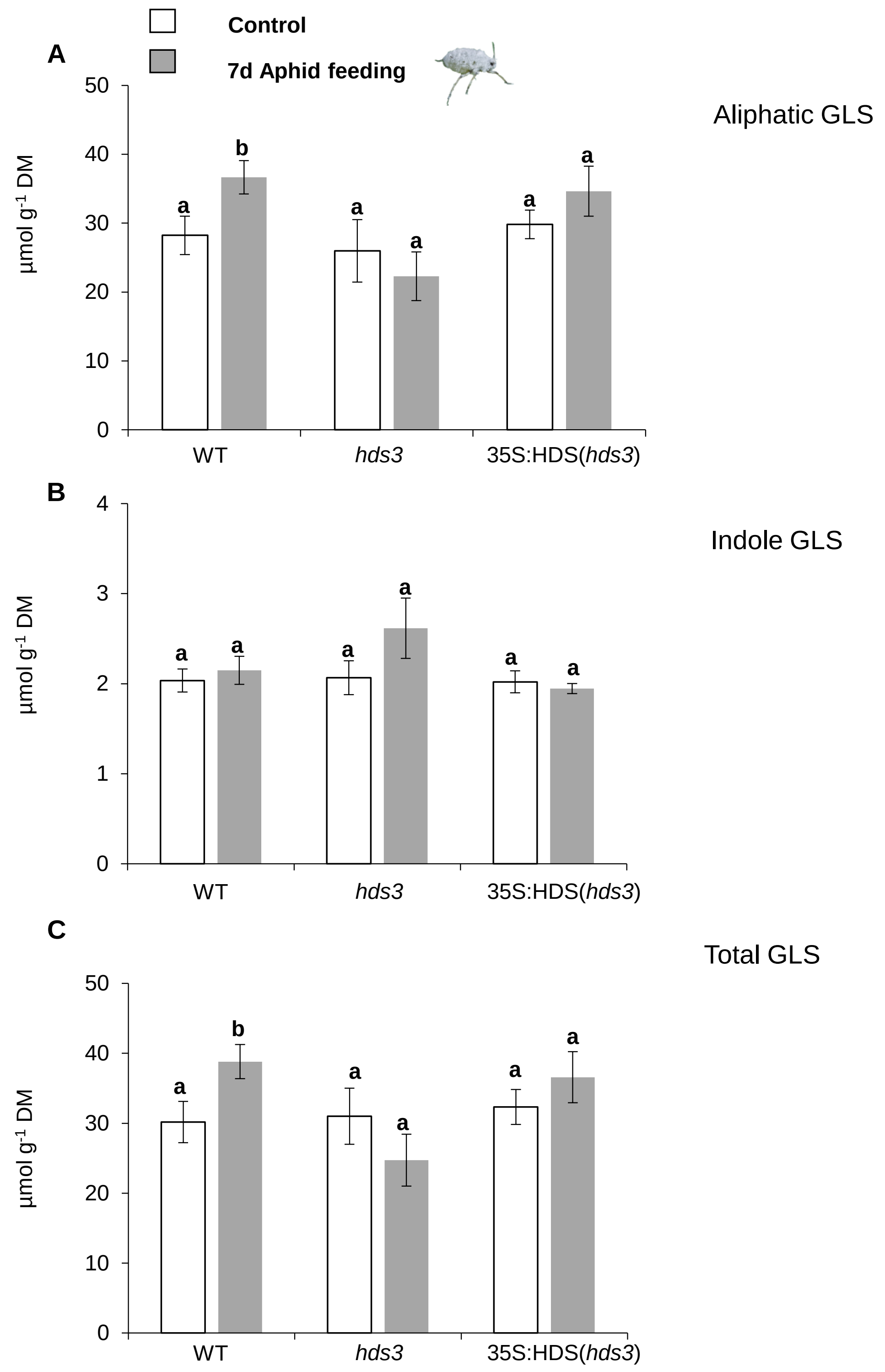

Control
7d Aphid feeding
A
Aliphatic GLS
µmol g-1 DM
50
40
30
20
10
0
a
b
a
a
a
a
WT
hds3
35S:HDS(hds3)
B
Indole GLS
4
3
2
1
0
a
a
a
a
a
a
C
Total GLS
a
b
a
a
a
a

Figure S4

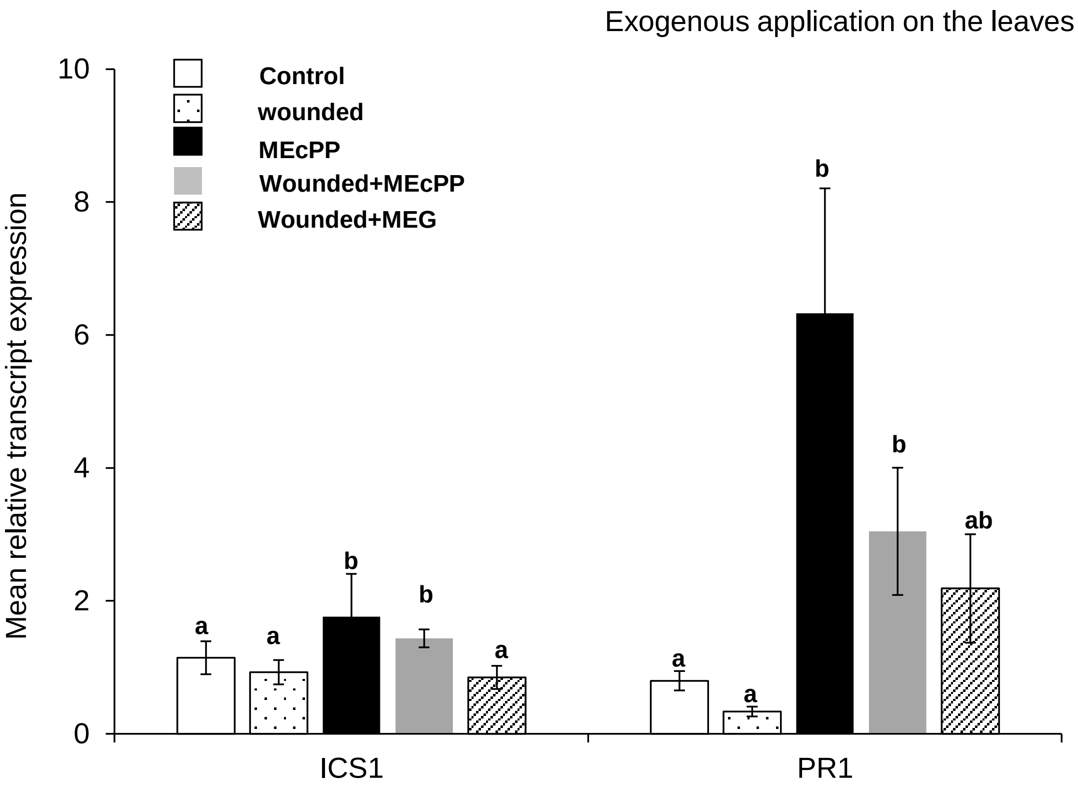
Exogenous application on the leaves
Mean relative transcript expression
Control
wounded
MEcPP
Wounded+MEcPP
Wounded+MEG
0
2
4
6
8
10
a
a
b
b
a
a
a
b
b
ab
ICS1
PR1

Figure S5

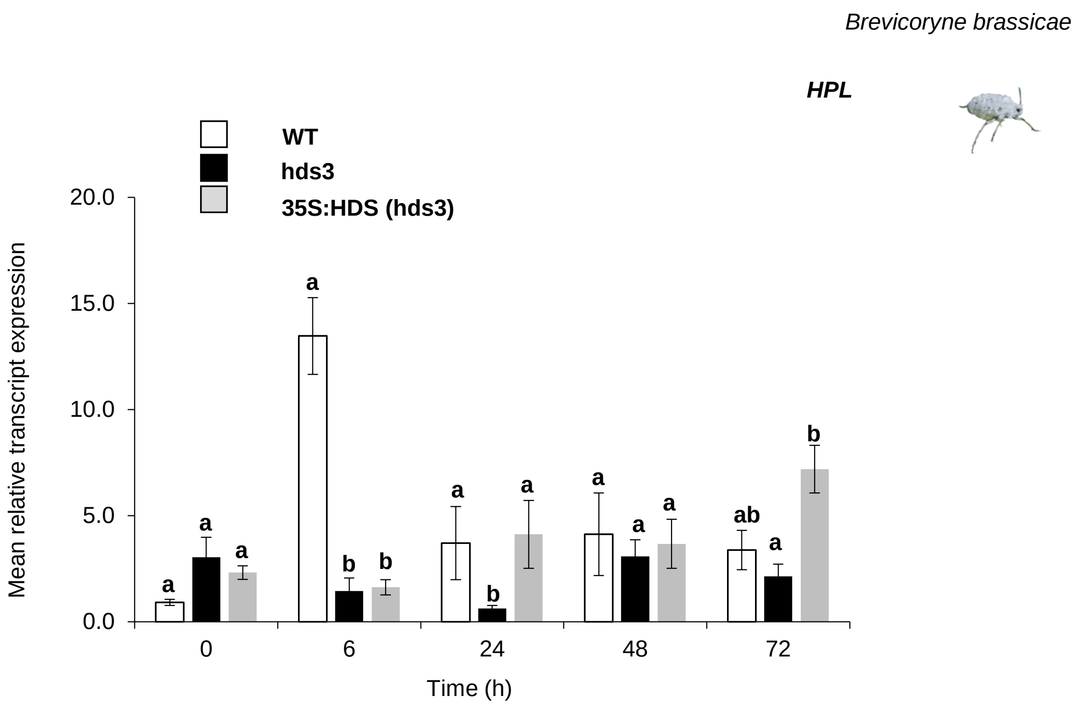
Brevicoryne brassicae
HPL
WT
hds3
35S:HDS (hds3)
Mean relative transcript expression
20.0
15.0
10.0
5.0
0.0
a
a
a
a
b
b
a
b
a
a
a
a
ab
a
b
0
6
24
48
72
Time (h)

Figure S6
